# Applications of Ballistocardiogram in the Diagnosis of Coronary Heart Disease: Systematic Review

**DOI:** 10.2196/68197

**Published:** 2025-08-08

**Authors:** Mairihaba Maimaiti, Hongling Zhu, Hesong Zeng

**Affiliations:** 1 Department of Internal Medicine, Tongji Hospital, Tongji Medical College, Huazhong University of Science and Technology, No. 1095 Jiefang Avenue, Wuhan, 430030, China, 86 13907199959

**Keywords:** ballistocardiogram, coronary heart disease, noninvasive, signal analysis, diagnostic performance, PRISMA

## Abstract

**Background:**

Coronary heart disease (CHD) continues to account for a substantial proportion of deaths worldwide. Ballistocardiogram (BCG), a noncontact, noninvasive technique for monitoring cardiac activity, has gained increasing attention for its potential role in various medical applications, particularly in CHD. This review comprehensively explores the applications of BCG in the diagnostic evaluation of CHD.

**Objective:**

The aim of this systematic review is to evaluate the clinical applications and diagnostic capabilities of BCG in CHD, with the ultimate goal of enhancing the precision of CHD management and optimizing therapeutic decision-making pathways.

**Methods:**

A literature search was performed in accordance with the PRISMA (Preferred Reporting Items for Systematic Reviews and Meta-Analyses) 2020 guidelines to identify studies evaluating the use of BCG in CHD. The initial search identified 500 studies. Based on titles, abstracts, and keywords, 266 studies were selected for further review. Following further exclusion of non-English articles, animal studies, and review articles, 38 eligible studies were included in the final analysis.

**Results:**

Among the 38 studies, 22 focused on the application of BCG in acute coronary syndrome. These studies explored various aspects, including BCG waveforms in patients with acute myocardial infarction, the diagnosis of acute coronary syndrome, and the relationship between age and the rate of abnormal BCG waveforms. The remaining studies covered the effects of drugs, emotions, exercise, and other variables on BCG recordings in patients with CHD. Sample sizes varied significantly across the studies, 36 studies explicitly reported sample sizes, encompassing a total of 9479 participants with individual study sizes ranging from 1 to 903 cases. Notably, 13 studies enrolled fewer than 50 participants, raising concerns about potential selection bias and reduced reliability of the findings.

**Conclusions:**

Overall, while BCG demonstrates significant potential in the diagnosis and prevention of CHD, several limitations remain. Variability in study design, sample size, and outcome measures poses challenges to the generalizability of findings. Nevertheless, the capability of BCG to reflect cardiac function and assist in the detection of CHD remains valuable. With continued research and technological advancement, BCG has the potential to transform current approaches to CHD diagnosis and management, ultimately improving patient outcomes and quality of life.

## Introduction

CHD remains a leading cause of global morbidity and mortality. It results from atherosclerosis and the subsequent narrowing or blockage of coronary arteries, leading to myocardial ischemia, hypoxia, or necrosis [1]. CHD may also involve other etiologies, such as inflammation and embolism, which can cause stenosis or occlusion of the vascular lumen. Despite the availability of diagnostic modalities, including electrocardiogram (ECG), exercise stress test, coronary computed tomography angiography, and coronary angiography, each method has inherent limitations. For instance, direct contact of ECG electrodes with the skin may cause allergic dermatitis. During acute myocardial infarction (MI) or severe arrhythmia, patients may be unable to perform the required exercise for the stress test. In addition, coronary computed tomography angiography involves a contrast agent injection, which may impair renal function or trigger allergic reactions, while also posing radiation risks. Although coronary angiography is the gold standard, it is invasive and costly. Consequently, developing a noninvasive, simple, and effective diagnostic method for CHD holds significant clinical value.

Research has definitively established that abnormal lipid metabolism is the primary pathogenic mechanism of CHD. Excess lipids accumulate on the arterial intima and penetrate the subendothelial space. Within the endothelium, these lipids are engulfed by macrophages, transforming into foam cells. As foam cells accumulate in significant numbers, they undergo apoptosis and necrosis, forming lipid necrosis cores. Over time, these cores evolve into atherosclerotic plaques, which gradually expand and narrow the vessels. When plaques rupture or ulcerate, thrombi may form, leading to blockage or severe stenosis of the coronary arteries [2].

BCG is a method that measures the force and velocity of the body’s recoil, resulting from the ejection of blood from the heart during each heartbeat. This recoil force, known as the ballistic force, is detected by sensors placed on the body surface, typically on the trunk or limbs. BCG captures and analyzes the mechanical vibration signals generated by cardiac activity to infer coronary artery stenosis. In a healthy state, myocardial contraction and relaxation are coordinated and powerful, producing stable and regular vibration signals. However, when coronary artery stenosis occurs, myocardial blood supply is reduced, impairing contraction and relaxation [3,4]. This dysfunction alters the mechanical vibration signals of the heart, manifesting as waveform abnormalities, reduced amplitude, or changes in frequency.

Compared with traditional methods, the key advantage of BCG lies in its noninvasive and straightforward nature, avoiding allergic reactions and invasive procedures. Furthermore, it enables real-time, continuous monitoring of cardiac function, allowing clinicians to assess patient status and treatment response with greater precision. However, to validate BCG’s value for CHD, further studies are needed to explore its correlation with biochemical indicators, such as blood lipid metabolism [5]. Such investigations will deepen our understanding of the potential and limitations of BCG in predicting coronary artery disease (CAD), thereby providing more accurate guidance for clinical diagnosis and patient management.

With the rapid advancement of artificial intelligence (AI) technologies, the processing and interpretation of BCG signals have also evolved toward more intelligent and automated approaches. Recent studies indicate that deep learning models, particularly convolutional neural networks and Siamese networks, offer promising solutions for feature extraction, anomaly detection, and disease assessment based on small-sample medical data [6,7]. These developments highlight the growing potential of integrating AI-integrated BCG analysis to enhance diagnostic accuracy and clinical utility.

This study systematically evaluates advancements and clinical applications of BCG specifically in CHD. We discuss BCG’s potential in diagnosing and monitoring CHD, focusing on its role in patients with and without acute coronary syndrome (ACS). Although BCG shows great promise, its clinical use is still fragmented and lacks unified validation. This study aims to clarify BCG’s diagnostic potential in CHD and explore how integrating AI technologies could improve diagnostic accuracy and expand its clinical applications.

The paper is structured as follows: Section 1 corresponds to the Introduction. Section 2 presents the Methods, focusing mainly on the search strategy. Section 3 contains the Results, which include the subsections: BCG in Detecting Medical Signals, BCG in ACS, and BCG in Non-ACS. Section 4 discusses the key findings and limitations of the study as well as the conclusions.

## Methods

### Literature Search

This review followed the PRISMA (Preferred Reporting Items for Systematic Reviews and Meta-Analyses) 2020 guidelines [8] and conducted a systematic search of English-language literature up to April 14, 2024, across PubMed, Scopus, Web of Science, and the Cochrane Library. The search strategy used keywords related to “ballistocardiogram,” “ballistocardiograms,” “ballistocardiograph,” “ballistocardiography,” “ballistocardiographic,” “BCG,” “coronary artery disease,” “coronary atherosclerotic heart disease,” “stable angina,” “unstable angina,” “ST segment elevation myocardial infarction,” “non-ST segment elevation myocardial infarction.” In addition, the reference lists of included studies were manually screened to identify further relevant literature. The full search strategies are provided in Multimedia Appendix 1.

### Selection Criteria

For the review, studies were included if they met the following criteria: (1) original research studies; (2) participants diagnosed with CHD; (3) research focus related to BCG; and (4) publication in English. Exclusion criteria were (1) non-English publications; (2) animal studies, reviews, or commentaries; (3) studies with unavailable full text; and (4) duplicate publications.

### Search Result

Studies were initially screened based on their titles, abstracts, and keywords, resulting in 266 potentially relevant studies. Of these, records were excluded due to inaccessible full texts (n=197), leaving records for full-text assessment (n=69). One reviewer performed the initial screening. For records with uncertain eligibility, a second reviewer was consulted to make the final inclusion decision. EndNote (Clarivate) was used for reference management, and no automation tools were applied in the screening process. Subsequently, non-English papers, reviews, and animal studies were excluded (n=17). Studies with duplicates were also removed (n=14). Finally, 38 studies [4,6,9-44] were included.

Data from the included studies were extracted by one reviewer. The extracted items included author and publication year, study content, sample size, and main findings. For any uncertainties during the extraction process, a second reviewer was consulted. No automation tools were used for data extraction, and the study authors were not contacted. EndNote was used solely for reference management. The extracted data are summarized in Multimedia Appendix 2.

Among the 38 studies [4,6,9-44], 22 [6,27,28,45-63] focused on the application of BCG in ACS. These studies explored various aspects, including BCG waveforms in patients with acute MI, the diagnosis of ACS, and the relationship between age and the incidence of abnormal BCG. The remaining studies covered the effects of drugs, emotions, exercise, and other variables on BCG recordings in patients with CHD.

The final selected studies were primarily conducted in the United States (n=27), the United Kingdom (n=4), China (n=3), Russia (n=1), and Sweden (n=3). Notably, the United States takes the lead, followed closely by the United Kingdom.

## Results

**Figure 1.**
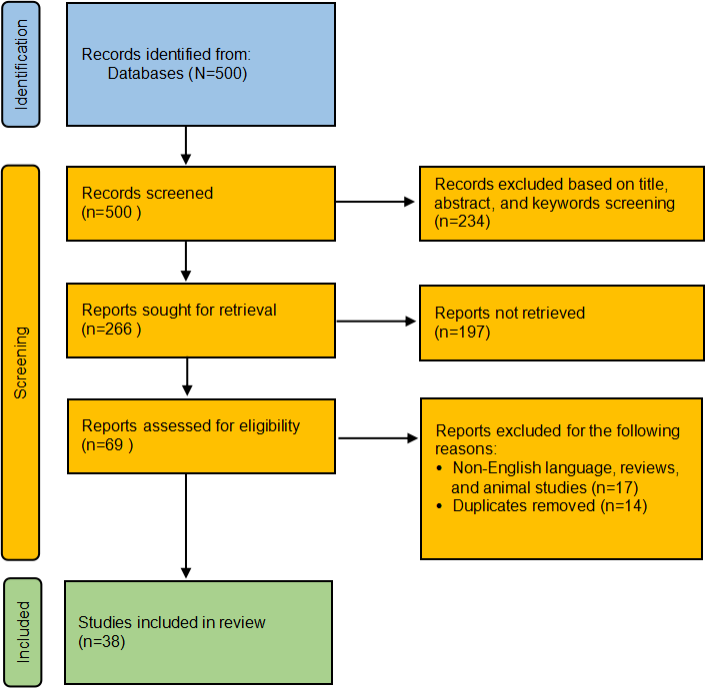
Preferred Reporting Items for Systematic Reviews and Meta-Analyses flow diagram.

The flow diagram illustrated in Figure 1 presents the study selection process based on inclusion and exclusion criteria. Before presenting the results of the included studies, we briefly introduce the general applications of BCG in medical signal detection.

### BCG in Detecting Medical Signals

Myocardial ischemia disrupts the heart’s conduction system, causing abnormal electrical impulses and arrhythmias, such as tachycardia or bradycardia. Coronary atherosclerosis narrows the lumen, potentially reducing or obstructing blood flow to distal areas, leading to ischemia or necrosis. To compensate, the heart increases its beat frequency to enhance cardiac output and alleviate inadequate perfusion. However, this compensatory tachycardia raises myocardial oxygen demand, worsening ischemia and perpetuating a vicious cycle. Hence, heart rate (HR) control is crucial in the management of CHD.

In HR monitoring, BCG signals have attracted considerable interest due to their rich physiological information. Unsupervised clustering algorithms [64-68] have been used to identify intrinsic patterns, while continuous wavelet transforms [69-74] effectively capture their time-frequency characteristics. Adaptive threshold algorithms [75-78] further enhance detection accuracy, and methods, such as the cepstral [45] and template matching [46,73], enhance HR feature extraction. The integration of machine learning has significantly advanced HR detection by enabling the automatic identification of relevant features from complex BCG signals, thereby expanding its clinical applications. Notably, reduced heart rate variability (HRV) due to autonomic dysfunction has been observed in cardiovascular conditions, such as myocardial ischemia, as well as in respiratory diseases like chronic obstructive pulmonary disease [47], highlighting the relevance of HRV analysis. In addition, the combination of beat-by-beat and BCG technology can effectively evaluate left ventricular function [9,10] and has the potential to become a noninvasive, low-cost left ventricular function assessment tool in the future. However, large-scale validation and rigorous methodological controls remain essential to establish its clinical reliability.

Autonomic neuropathy and vascular dysfunction frequently coexist and influence each other’s manifestations. In cases where coronary artery stenosis results in myocardial ischemic necrosis, the damaged myocardium stimulates chemical and mechanical receptors on the cardiac wall, triggering a sympathetic stress response. This heightened sympathetic activity reduces HRV [48], thereby increasing the risk of cardiac instability. Therefore, effective management of CHD requires an integrated approach that addresses both autonomic and vascular components to optimize cardiac function.

The sophistication of BCG signals for HRV detection has enabled diverse applications. Sensors like ElectroMechanical Film [49-51] and Polyvinylidene Fluoride [53-55], commonly embedded in seat belts and smart beds [51,56,79], are widely used to capture BCG signals for HRV assessment. In addition, physiological signals can be recorded using data acquisition systems such as BIOPAC (BIOPAC Systems) [52]. These technologies are adaptable to various environments, expanding the scope of HRV monitoring. Comparative studies highlight BCG’s utility, showing strong agreement between BCG and ECG in HRV measurements and revealing consistent trends [50]. This concordance positions BCG as a valuable complement to ECG, enhancing the comprehensiveness of HRV assessment. With ongoing technological advances and further research, the applications of BCG in HRV detection are expected to expand. Its versatility and reliability in various settings promise a bright future for BCG in HRV detection. Meanwhile, studies on wearable blood pressure (BP) monitoring, such as those using cuffless pulse transit time (PTT) methods, have demonstrated promising accuracy and usability, indicating the growing potential of wearable technologies in cardiovascular health monitoring [57].

Patients with CHD often exhibit complex and variable BP patterns. Before the onset of cardiac symptoms, BP may elevate, reflecting reduced cardiac compliance—diminished heart elasticity in response to blood flow, necessitating higher pressures for normal circulation [58]. However, during acute attacks, ischemia can significantly reduce myocardial contractile force, causing BP to drop. Compounding this, many patients also have hypertension, a chronic condition that not only accelerates coronary atherosclerosis but also chronically overloads the heart, further compromising myocardial function. This dual disease burden significantly raises the risk of cardiovascular events, namely MI and heart failure.

BCG has emerged as a standout in BP detection, leveraging its unique characteristics. The signal, generated by cardiac pulsations and arterial blood flow, represents changes in the external body pressure, enabling noncontact monitoring of heart activity. BCG, when combined with methods such as PTT or photoplethysmography, shows comparable accuracy to traditional cuff-based blood pressure devices. For instance, one study reported root mean square errors of 6.7 (SD 1.6) mmHg for systolic and 4.8 (SD 1.5) mmHg for diastolic BP, similar to values from standard sphygmomanometry [59,60]. By using techniques, such as clustering [61], regression analysis [62], and deep neural networks [80], key metrics like pulse arrival time [50,62,81] and PTT [63,82-91] can be accurately calculated to estimate BP. In addition, technological advancements are driving innovations in BCG-based BP monitoring methods. These include novel devices, such as chair-based systems [92,93] and wearable limb BCG [87], broadening the possibilities for accurate and convenient BP monitoring.

In summary, BCG offers distinctive advantages in detecting vital indicators. Its noninvasive and user-friendly nature positions it as a pivotal tool for cardiac function assessment, affording doctors greater precision and depth in diagnostic analysis.

### BCG in ACS

For patients with MI, especially those with non-ST-segment elevation MI and ST-segment elevation MI, the application of BCG technology holds significant importance. During an MI, myocardial cell necrosis and injury affect the heart’s mechanical and electrical activities, leading to subtle waveform changes in BCG recordings. For instance, features such as a low I wave, deep K wave, high H wave, and a notched J wave can all serve as important indicators of MI [11-16]. Moreover, abnormal waveforms and amplitude changes are more pronounced on the acceleration curve than on the displacement or velocity curve [17]. A study [18] conducted thorough ECG and BCG measurements on 78 patients with angina pectoris and 32 patients with distal MI. The analysis revealed that 40 (36%) cases of patients exhibited abnormal ECGs, a concerning figure indeed. However, the most startling finding was that 88 (80%) cases showed abnormal BCG results, indicating high sensitivity of this diagnostic tool in detecting heart diseases. Furthermore, they discovered that 94 (85%) patients exhibited at least 1 or 2 abnormalities in either their ECGs or BCG, emphasizing the intricate and diverse nature of heart disease manifestations. Research [19-21] has shown that abnormal BCG can be recorded in MI. Concurrently, in one observational study [94], all patients with symptomatic MI exhibited BCG abnormalities, further demonstrating its potential in MI detection [30].

With increasing age, the incidence of abnormal ECG and BCG findings among older patients with MI shows a pronounced upward trend [22]. Specifically, the frequency of such abnormalities increases significantly with each decade, particularly when compared to age-matched healthy individuals. This significant finding underscores the unique cardiac function changes in older patients with MI, holding immense importance for the prevention and early detection of this condition. Studies have reported that clinically healthy individuals under 40 years of age rarely exhibit abnormal BCG recordings. However, after the age of 80 years, the occurrence of abnormal electrocardiographic findings rises sharply, approaching 100% in certain cohorts [23]. This escalating trend underscores the deterioration of central organ function and the escalating risk of heart disease among the older population, emphasizing the need for vigilant monitoring and proactive management. Cardiovascular aging is a natural phenomenon that involves the gradual deterioration of vascular structure and function as a person ages [95]. A study used BCG to observe 21 patients under 54 years old with a history of MI. They found that 17 (81%) cases exhibited abnormal BCG results, directly reflecting accelerated cardiovascular aging, particularly a significant decrease in HJ force (the force of the H and J waves of the BCG) [24]. This abnormality rate was significantly higher than that in age-matched healthy controls, indicating signs of accelerated cardiovascular aging. These findings suggest that BCG-derived metrics may serve as early indicators of subclinical ischemic heart disease (IHD), allowing for earlier risk stratification and preventive intervention in at-risk populations.

Portable BCG monitoring has demonstrated potential as a diagnostic tool for detecting CAD in patients with angina pectoris [25]. In cases of insufficient coronary artery blood supply, there is a high incidence of abnormal BCG patterns, with the deep K stroke pattern occurring frequently. A parallel study [5] investigated the correlation between BCG and MI, as well as the interplay with blood lipids. The findings suggested that BCG may serve as an indicator of abnormalities in individuals predisposed to MI and that BCG abnormalities may be influenced by factors, such as smoking, physical exertion, and emotional state. Meanwhile, BCG analysis incorporating respiratory monitoring successfully identified left ventricular dysfunction beats in a United States cohort study [10]. Using a nonlinear quadratic discriminant function, the study discovered that 87% (239/275) of the heartbeats in healthy males were classified as “normal,” while 98% (45/46) of the heartbeats in males with CHD were accurately identified as “coronary heart” beats. Building on prior research, a beat-by-beat analysis was conducted to assess left ventricular contractility and abnormal beats, ultimately discovering that 96% of heartbeats stemming from 6 patients with MI were accurately categorized as resembling “CAD-like” heartbeats [9], indicating the promising role of BCG in cardiac abnormality detection.

To better quantify and assess the severity of abnormalities in BCG after MI, some studies have introduced a grading system [16]. This system divides BCG into 4 grades: grade I represents minimal abnormalities, indicating that the patient’s heart function is basically normal; grade II indicates moderate abnormalities, suggesting that the patient’s heart function has recovered to some extent but is not yet fully normalized; grade III represents significant abnormalities, indicating severe damage to the heart with limited recovery; and grade IV represents the most severe abnormalities, suggesting severe impairment and poor recovery of heart function. This grading system provides clinicians a systematic framework for severity stratification, thereby informing more personalized and targeted treatment strategies.

BCG is not only used for the diagnosis of MI but also effectively assesses the treatment outcomes and prognosis of patients [26]. Research has shown that the prediction of subsequent MI or sudden death by BCG is highly accurate, with a statistical significance of *P*<.001 [27]. By comparing changes in BCG signals before and after treatment, clinicians can visually assess the recovery of patients’ heart function, predict potential risks of complications, and accordingly adjust and optimize treatment plans. With its real-time monitoring, high sensitivity, and specificity, BCG provides powerful support for the diagnosis and treatment of patients with MI.

### BCG in Non-ACS

A study using low-frequency BCG to assess cardiac function in patients with atherosclerotic heart disease found that, although IJ amplitude (the amplitude difference between the I wave and the J wave in the BCG signal) exhibited respiratory-related fluctuations in these patients, the changes were not significantly different from those observed in age-matched healthy individuals [28]. This finding contributes to our understanding of the physiological implications of arterial sclerosis, particularly regarding IJ amplitude variability. However, a pivotal observation emerged from their study. In cases where therapeutic interventions fail to arrest disease progression, a discernible upward trend in IJ amplitude becomes evident. This telling shift may serve as a harbinger of waning heart function or exacerbating pathological insults, thereby providing clinicians with a vital tool for evaluating treatment responsiveness and tracking disease trajectories.

Recent studies have expanded BCG’s diagnostic applications in cardiovascular assessment. Analysis of myocardial functional integrity [29] demonstrated particularly high clinical utility for life insurance evaluations. The research underscored the heightened prevalence of BCG abnormalities among the older populations. Subsequent studies [3] further validated BCG’s diagnostic prowess by using it to screen for suspected CHD in individuals with chronic chest pain, contrasting results with ECG findings. Among 197 patients with CHD, 159 (81%) exhibited an abnormal BCG, while only 14 (7%) cases presented with concurrent normal BCG and ECG readings, underscoring BCG’s diagnostic sensitivity. In another investigation, comparative analysis of BCG recordings from patients with IHD and healthy participants revealed robust correlations between IJ velocity, vanillylmandelic acid excess, and cardiovascular risk. Specifically, patients with IHD on the brink of mortality consistently exhibit lower initial IJ amplitudes, offering invaluable insights into the prognostic value of BCG in assessing imminent cardiac events.

Noninvasive BCG assessment achieved a diagnostic accuracy of 77%, correctly stratifying 289 of 375 patients with CHD severity when validated against the gold standard of coronary angiography [31]. This result highlights BCG’s promise as a diagnostic adjunct in cardiovascular assessment. A study [32] used BCG technology to monitor HR and respiration in the 3 months following coronary artery bypass grafting surgery. The findings revealed that the mean respiration rate was 21.8 (SD 2.5) breaths per minute, while the mean HR was 67.6 (SD 2.4) beats per minute. These findings underscore the stability and recovery trajectory of patients undergoing this complex surgical procedure. Building upon these successes, other researchers used BCG to assess myocardial contractility and prognosis before and after coronary artery bypass grafting [33]. Their findings indicated a noteworthy 3% increase in average HR and myocardial strength following the surgical intervention. This improvement not only validates the efficacy of the surgical intervention but also highlights the potential of BCG as a sensitive tool for assessing cardiac function and predicting patient outcomes.

An observational study compared the BCG of 77 patients with CHD and 48 healthy individuals and further analyzed the changes in BCG parameters, such as time interval from I wave to J wave, time interval from J wave to K wave, and energy of the HIJK wave complex, before and after surgery [34]. Notably, statistical analysis revealed significant differences across these indicators: time interval from I wave to J wave (mean 95, SD 9 vs mean 78, SD 11 ms), time interval from J wave to K (mean 72, SD 10 vs mean 63, SD 8 ms), and energy of the HIJK wave complex (mean 0.020, SD 0.009 vs mean 0.010, SD 0.006 V²). Postoperatively, the BCG amplitude exhibited an increase, and the I-peak became significantly deeper, suggesting positive physiological changes. Also, a novel method was developed using micromotion-sensitive mattresses to gather BCG signals and then leveraged the ensemble empirical mode decomposition method to calculate HRV for disease classification [35]. This approach achieved a diagnostic accuracy of 92% by correctly classifying 17 of 18 participants. These results highlight the value of integrating advanced signal acquisition and processing in BCG-based diagnostics. Further advancing the field, another study [36] used the combination of short-time Fourier transform and ensemble empirical mode decomposition to accurately identify IJK complexes within BCG signals and assess HR. The proposed approach achieved a mean absolute error of 0.99 (95% CI −1.81 to 3.79) bpm, underscoring the high degree of accuracy and reliability achieved through the fusion of these advanced analytical techniques, opening new avenues for BCG-based cardiac monitoring and diagnosis.

The research has revealed that inducing hypoxemia serves as an advantageous approach in assessing critical physiological indicators like cardiac output and pulse pressure in humans under stressful conditions. This technique enables the maintenance of a consistent stress level for an extended period, ultimately ensuring the reliability and precision of collected data. Critically, the induction of hypoxemia minimizes the likelihood of artificial distortions in BCG recordings, a pivotal factor in securing accurate diagnostic outcomes [96]. As a result, when ECG and BCG readings in the resting state are inconclusive or ambiguous [37], it is advisable to consider capturing these 2 data types again in a simulated hypoxemic environment. This strategy may yield more profound and definitive diagnostic insights into CAD [38], particularly in scenarios where traditional diagnostic methods fail to provide clear-cut results. The value of this approach cannot be overstated.

As is well known, smoking history is one of the risk factors for CHD. Smoking damages endothelium and promotes atherosclerosis. Notably, smoking can also significantly alter the BCG of patients [39]. Studies have highlighted a stark contrast, with only approximately 8/114 (6.8%) healthy individuals experiencing BCG deterioration post smoking, compared to a staggering 51/86 (59%) individuals with CHD [40]. To further explore the intricate interplay between emotional states and BCG dynamics, BCG monitoring was performed on 48 patients with IHD [41], aiming to elucidate the association between emotional states and alterations in IJ velocity. The analysis demonstrated that 5/6 participants exhibited a positive correlation between IJ velocity and emotional arousal (*r*=0.34‐0.90), while 4/6 participants displayed a positive correlation between HR and emotional arousal (*r*=0.60‐0.90). These findings underscore the intricate link between emotional states and BCG parameters, particularly in patients with IHD, highlighting the potential clinical significance of monitoring BCG in such contexts.

Excessive exercise load can significantly exacerbate symptoms in patients with CHD and even trigger severe angina. In this emergency situation, nitroglycerin becomes the key medication to alleviate symptoms. According to research results, the majority of patients with CHD showed significant improvement in their BCG after taking nitroglycerin [13,42]. Nitrite also has an impact on the BCG. After inhaling amyl nitrite, healthy individuals may experience a temporary increase in BCG amplitude. In patients with CAD, this change is minimal. Moreover, the IJ waveform of BCG cannot accurately reflect the cardiac output [43]. BCG monitoring of isosorbide dinitrate therapy in patients with CHD demonstrated that normalization of BCG was more prevalent among patients experiencing symptom improvement compared to those with normal ECG [44]. This suggests that BCG provides a more sensitive and objective evaluation tool than ECG for assessing therapeutic effects in patients with CHD.

By continuously monitoring changes in heart electrical activity, BCG technology provides timely diagnostic evidence for doctors, enabling them to quickly adopt emergency treatment measures, effectively preventing further deterioration of the condition, and buying precious treatment time for patients.

## Discussion

### Principal Findings

This study provides a comprehensive review and analysis of the widespread application of BCG in the field of CHD and its use in monitoring vital signs, emphasizing its importance and future potential. BCG, as a novel technology for cardiovascular function monitoring, uses relevant techniques to detect key indicators such as HR, HRV, and BP. It not only offers a new method for HR monitoring but also represents a low-cost, high-precision, and noncontact technology. In CHD, BCG aids in diagnosis and disease classification, and it plays a critical role in assessing treatment effectiveness. According to the literature, 208/239 (87%) heartbeats from healthy males were correctly classified as “normal” through BCG analysis, while 45/46 (98%) heartbeats from male patients with CHD were accurately identified as “CHD” heartbeats. When compared to coronary angiography, BCG showed a 77% accuracy by categorizing 289/375 patients according to their CHD severity. The application of BCG technology not only improves the accuracy and efficiency of diagnosis but also significantly enhances targeted and personalized treatment in patient care, revolutionizing the management and treatment of CHD.

### Limitations

In terms of the review process, several limitations should be noted. First, although we adopted a systematic approach, only English-language literature was included, which may introduce language bias. Second, data extraction was performed by a single reviewer, with a second reviewer consulted only when uncertainties arose; this could lead to potential subjectivity. Furthermore, no formal quality assessment of the included studies was conducted, and the review protocol was not prospectively registered, which may affect the transparency and reproducibility of the review.

Although BCG has become a research hotspot due to its potential and prospects in the medical field, its application in CHD research is still relatively limited. Most of the studies are concentrated in high-income countries, such as the United States and the United Kingdom. However, the total number of related studies remains small globally, indicating that the research activity and depth in this field remain insufficient. Although some studies have reported sample sizes exceeding 900, this number still appears relatively small compared to other more mature research fields, indicating that large-scale studies based on BCG are still significantly insufficient in CHD research. In terms of research content, the current focus is mainly on the analysis of waveform abnormalities, and there is a lack of in-depth research and clear conclusions on how to specifically use BCG to diagnose CHD, as well as which specific waveform changes correspond to CHD. This has limited the application of BCG technology in the diagnosis of CHD. As for accuracy, although BCG technology has shown an acceptable level of accuracy in current research, it is important to acknowledge that sensor design and signal processing itself is a complex and highly specialized field. These factors may all have an impact on the accuracy and reliability of BCG. Therefore, more in-depth and systematic research and validation are needed before applying it to the diagnosis of CHD. To improve BCG accuracy in CHD research, identifying and removing artifacts is crucial. Recent studies have highlighted the importance of multimodal monitoring, particularly the integration of ECG and BCG, for artifact reduction, offering valuable insights for BCG’s CHD application [97]. In addition, in home environments, BCG signals may be affected by data loss and motion artifacts due to patient movement or device misplacement, further affecting signal quality and diagnostic reliability [47]. In summary, although BCG has shown certain potential and prospects, there are still many challenges and limitations in its application in the diagnosis of CHD. Therefore, further research and development are required to better explore the potential of BCG and to establish a more robust and reliable theoretical and practical foundation for its application in the diagnosis of CHD. While this review focuses on the current clinical applications and challenges of BCG in CHD, the rapid advancement of AI presents an exciting opportunity to enhance BCG signal analysis. Future studies should investigate AI-driven feature extraction and predictive modeling to improve diagnostic accuracy and facilitate personalized cardiovascular monitoring.

### Conclusions

BCG is a noninvasive cardiovascular detection method. Through summarizing relevant literature, the application of BCG in CHD has been explored, mainly in recording BCG in patients with MI and angina pectoris. These studies have laid the foundation for the diagnosis and treatment of CHD in the future. With the continuous innovation and improvement of technology, BCG is expected to play an increasingly important role in the diagnosis and treatment of CHD. Its unique advantages and potential make it an important pillar in this field, bringing more hope and good news to patients. It is anticipated that as research deepens and technology matures, BCG will be able to diagnose more accurately, providing doctors with more comprehensive and accurate patient information, which will facilitate the development of personalized treatment plans. This will help improve the treatment effectiveness of CHD, enhance patients’ quality of life, and even potentially reduce medical costs, bringing greater benefits to society. At the same time, it is hoped that more researchers will contribute to the development of BCG, advancing progress and innovation in this field. Through interdisciplinary collaboration, further exploration of the potential of BCG in CHD diagnosis and treatment can be achieved. This will not only help promote the advancement of medical technology but also bring more blessings and hope to patients. In short, as an emerging technology, BCG has unlimited possibilities and hopes for application in the field of CHD. It is expected to improve treatment outcomes and quality of life for patients, while playing an increasingly important role in the development of the medical field.

## Supplementary material

10.2196/68197 Multimedia Appendix 1 Complete search strategies for all databases.

10.2196/68197 Multimedia Appendix 2 Summary of included studies in the review.

10.2196/68197 Checklist 1 PRISMA checklist

## Abbreviations

ACS: acute coronary syndrome
AI: artificial intelligence
BCG: ballistocardiogram
BP: blood pressure
CAD: coronary artery disease
CHD: coronary heart disease
ECG: electrocardiogram
HR: heart rate
HRV: heart rate variability
IHD: ischemic heart disease
MI: myocardial infarction
PRISMA: Preferred Reporting Items for Systematic Reviews and Meta-Analyses
PTT: pulse transit time
