# Supplementary material for: Applications of Ballistocardiogram in the Diagnosis of Coronary Heart Disease: Systematic Review
Source: JMIR Cardio. 2025 Aug 8;9:e68197. doi: 10.2196/68197 (PMC12334112; doi:10.2196/68197)
Supplement: Multimedia Appendix 1 [file cardio-v9-e68197-s001.doc]

Multimedia Appendix 1. Search Strategy

PubMed:

Date of Search: from 1950 to April 14, 2024

((BCG) OR (ballistocardiogram) OR (ballistocardiograms) OR (ballistocardiograph) OR (ballistocardiography) OR (ballistocardiographic)) AND (( stable angina) OR (unstable angina) OR (ST segment elevation myocardial infarction) OR (non-ST segment elevation myocardial infarction) OR (coronary artery disease) OR (coronary atherosclerotic heart disease))

Scopus：

Date of Search: from 1950 to April 14, 2024

TITLE-ABS-KEY(((BCG) OR (ballistocardiogram) OR (ballistocardiograms) OR (ballistocardiograph) OR (ballistocardiography) OR (ballistocardiographic)) AND (( stable angina) OR (unstable angina) OR (ST segment elevation myocardial infarction) OR (non-ST segment elevation myocardial infarction) OR (coronary artery disease) OR (coronary atherosclerotic heart disease)))

Web of Science：

Date of Search: from 1950 to April 14, 2024

(TS=((BCG) OR (ballistocardiogram) OR (ballistocardiograms) OR (ballistocardiograph) OR (ballistocardiography) OR (ballistocardiographic))) AND TS=(( stable angina) OR (unstable angina) OR (ST segment elevation myocardial infarction) OR (non-ST segment elevation myocardial infarction) OR (coronary artery disease) OR (coronary atherosclerotic heart disease))

Cochrane：

Date of Search: from 1950 to April 14, 2024

 ((BCG) OR (ballistocardiogram) OR (ballistocardiograms) OR (ballistocardiograph) OR (ballistocardiography) OR (ballistocardiographic)) AND (( stable angina) OR (unstable angina) OR (ST segment elevation myocardial infarction) OR (non-ST segment elevation myocardial infarction) OR (coronary artery disease) OR (coronary atherosclerotic heart disease)) in Title Abstract Keyword - (Word variations have been searched)
