# Supplementary material for: Applications of Ballistocardiogram in the Diagnosis of Coronary Heart Disease: Systematic Review
Source: JMIR Cardio. 2025 Aug 8;9:e68197. doi: 10.2196/68197 (PMC12334112; doi:10.2196/68197)
Supplement: Multimedia Appendix 2 [file cardio-v9-e68197-s002.doc]

## Summary of Included Studies in the Review

| Author | Year | Country | Content | Sample | Result |
| --- | --- | --- | --- | --- | --- |
| Baker, B.M.[18] | 1950 | USA | Comparing BCG^a^ between normal individuals and patients with MI^b^ | 110 subjects | 80% of patients exhibit abnormal BCG results |
| W R SCARBOROUGH[38] | 1951 | USA | Comparing ECG^c^ and BCG during hypoxia in patients with CAD^d^ | 27 subjects | one patient's ECG was normal, another showed abnormalities, and some BCGs became more abnormal during hypoxia. |
| Mandelbaum, H.[25] | 1951 | USA | BCG analysis for hypertension, normal, CAD conditions. | 268 subjects | Poor coronary supply, frequent deep K patterns. |
| Jones, R. J.[28] | 1952 | UK | Compared IJ amplitude and wave timing in patients with ASHD^e^ and CHF^f^ | 121 subjects | Age and HF increase IJ abnormalities; amplitude rises after diuresis |
| Taymor, R. C.[21] | 1952 | USA | Exercise ECG and BCG to assess circulatory responses in CAD diagnosis | 195 subjects | Negative "Two-Step": 27% abnormal BCG; Positive "Two-Step": 93% abnormal; Borderline "Two-Step": 6/7 abnormal |
| Harry Mandelbaum[42] | 1952 | USA | Changes in BCG after taking nitroglycerin in angina pectoris | 2 subjects | Significant improvement in BCG after nitroglycerin use |
| Scarborough, W. R.[22] | 1952 | USA | Comparison of BCG and ECG between MI and normal individuals | 328 subjects | 69% of old MI had abnormal ECGs, 72% had abnormal BCGs. 24% of angina patients had abnormal ECGs, while 75% had abnormal BCGs. |
| Moser, M.[11] | 1952 | USA | Observing changes in BCG after rest and exercise in MI patients | 100 subjects | Low I Wave:21 cases; bizarre pattern:18 cases; a notched J wave:15 cases; a prominent H wave:12 cases; Low I and Notched J:7 cases; Low Voltage:6 cases; Low I deep K:2 cases |
| Davis Jr, F. W.[40] | 1953 | USA | Comparison of BCG changes after smoking and exercise in CAD | 200 subjects | 6.8% of normal and 58.6% of CAD patients showed worsened BCG after smoking. 10 subjects showed similar changes with 0.5mg nicotine |
| J E SMITH[17] | 1953 | USA | Observing BCG in MI patients of different ages | 20 subjects | The JK/IJ ratio for 35-year-old women is low and varies by age and gender. |
| Henderson, C. B.[39] | 1953 | UK | Observing the effects of smoking on cardiovascular dynamics in CHD^g^ using BCG | 124 subjects | 4 older showed temp BCG abnormalities after smoking. 15 CHD showed BCG changes post-smoking. |
| Harry E. Ungerleider[29] | 1953 | USA | Evaluating cardiac function using BCG in patients with CAD | - | The abnormality rate of BCG is higher in elderly individuals |
| Mandelbaum, H.[16] | 1953 | USA | Observing BCG to evaluate the recovery of myocardial function in hypertensive and CAD | 100 subjects | 65 patients: improved to grade II+; 1 patient: advanced to grade III post-2nd MI; 35 patients: advanced abnormalities post-MI |
| H D JACOBS[43] | 1954 | UK | Observing the effect of inhaling amyl nitrite on the IJ wave of BCG in CAD patients | 75 subjects | Amyl nitrite inhalation briefly boosts BCG amplitude in normals, but minimal in CAD patients. |
| Rivin, A. U.[19] | 1954 | USA | Measurement of BCG in patients with hypertensive and CAD | 225 subjects | Hypertension deepens K-waves. In hypertensive heart disease, K wave deepens and IJ wave decreases. Asymptomatic CHD with normal ECG may show abnormal BCG. |
| Starr, I.[13] | 1955 | USA | Giving nitroglycerine to observe BCG changes in CHD | 111 subjects | Amyl nitrite increased HR^h^ and BCG amplitude; nitroglycerin lowered BP |
| Kuo, P. T.[12] | 1955 | USA | Giving angina pectoris patients fatty meal and recording serial ECG and BCG | 14 subjects | 12 out of 14 patients (86%) had severely abnormal BCG |
| Wade, E. G.[3] | 1956 | UK | Comparison of BCG and ECG between CAD and normal individuals | 235 subjects | BCG was abnormal in 159 out of 196 cases (81.1%) |
| Isaacs, J.[14] | 1958 | USA | Comparison of BCG between MI  and normal individuals | 168 subjects | 22 of 30 subjects (73%) had wide H waves; 23 of 30 subjects (77%) had tall H waves; N wave was very prominent in 20 of 30 subjects (67%) |
| SMITH DH[5] | 1959 | USA | The relationship between BCG, MI, and blood lipids | - | BCG alone may indicate MI predisposition. Combining BCG and cholesterol improves prediction accuracy |
| Scarborough, Wm R.[23] | 1960 | USA | relationship between lipid levels and BCG abnormalities in patients with CHD | 280 subjects | BCG was normal in those under 40, but abnormalities reached 100% in the 8th decade |
| Moss, A. J.[24] | 1962 | USA | Analysis of BCG on Cardiovascular Function in CHD patients | 21 subjects | 17 out of 21 patients (81%) had abnormal BCG indicative of accelerated cardiovascular aging |
| Richman, S. M.[37] | 1963 | USA | the reliability and effectiveness of BCG in CHD | 63 subjects | 21 out of 32 patients (66%) had abnormal BCG |
| W K Harrison Jr[20] | 1967 | USA | Comparison of cardiac function between normal individuals and patients with CHD | 44 subjects | 33 out of 44 subjects (75%) were correctly classified by the discriminant analysis |
| T N Lynn[27] | 1974 | USA | Observing BCG in normal individuals and MI | 134 subjects | highly accurate with p< .001 |
| Theorell, T.[41] | 1974 | Sweden | Calculating IJ velocity, heart rate, and their correlation with emotional states in IHD^i^ patients | 48 subjects | 5 out of 6 subjects (83%) showed a positive IJ-emotion correlation (r= 0.34–0.90). 4 out of 6 subjects (67%) showed a positive HR-emotion correlation (r= 0.60–0.90). |
| T Theorell[26] | 1975 | Sweden | Observation of the relationship between BCG and prognosis in MI patients | 36 subjects | The peak amplitude of the I-J wave in the last six months was significantly higher than in the previous periods (F=4.43) |
| Starr, I.[33] | 1975 | USA | Cardiac contractility before and after coronary artery bypass grafting in CHD patients | 100 subjects | 3 out of 100 patients (3%) died；Significant increase in average HR and strength after surgery |
| Tores Theorell[30] | 1975 | USA | Analyzed IJ amplitude and velocity in relation to 24-hour urinary VMA^j^ levels, MI, and sudden death risk | 123 subjects | IJ velocity correlated with VMA and MI risk; IJ amplitude remained low in IHD deaths |
| Trakhtenberg Sb[44] | 1976 | Russia | BCG in CAD patients treated with ISDN^k^ | 120 subjects | Symptom improvement; BCG normalization more sensitive than ECG |
| William H Bancroft[31] | 1976 | USA | Assess CAD presence and severity using BCG parameters | 903 subjects | 695 out of 903 patients (77.0%) were correctly classified by the regression model |
| De Faire, U.[15] | 1979 | Sweden | Evaluating cardiac function and its relationship with IHD | 31 subjects | coefficient of 0.82 |
| Dinaburg, A. G.[10] | 1984 | USA | Recording STI^l^, BCG, and respiratory cycles, then applying discriminant analysis to detect LVF^m^ dysfunction in MI and hypertensive patients | 222 subjects | 87% of normal males had "normal" heartbeats, while 98% of males with coronary heart disease had "coronary" heartbeats |
| Dinaburg, A. G.[9] | 1987 | USA | Left ventricular contractility beat-by-beat analysis and its correlation with MI | 23 subjects | 96% of beats from 6 MI men were classified as "coronary-like." |
| Xinrong CAO[34] | 2014 | China | Comparison of preoperative and postoperative BCG in CHD patients | 125 subjects | Significant differences in tIJ, tJK, IntHK, and IntBCG (p<.05). Postoperative increase in BCG amplitude and deepened peak I |
| Yalong Song[35] | 2015 | China | SET-EMD^n^ analyzes BCG signals, calculates HRV^o^ parameters, and classifies normal, hypertension, and CHD patients | 18 subjects | Out of 18 subjects, the classification accuracy achieved was 92.3% |
| Nicholas Harrington[32] | 2021 | USA | Non-contact sensor under the bed monitors HR, RR^p^, and BCG in heart disease patients | 1 subject | Mean RR was 21.8 (SD^q^ 2.5); mean HR was 67.6 (SD 2.4) |
| Jingda Feng[36] | 2023 | China | Using STFT^r^ and SET-EMD to identify IJK complex waves in CHD patients | 12 subjects | MAE^s^ was 0.99 bpm (95% CI^t^: ± 2.8 bpm) |

^a^BCG: Ballistocardiogram

^b^MI: myocardial infarction

^c^ECG: Electrocardiogram

^d^CAD: coronary artery disease

^e^ASHD: arteriosclerotic heart disease

^f^CHF: congestive heart failure

^g^CHD: Coronary heart disease

^h^HR: heart rate

^i^IHD: ischemic heart disease

^j^VMA: Vanillylmandelic Acid

^k^ISDN: isosorbide dinitrate

^l^STI: Systolic Time Interval

^m^LVF: left ventricular function

^n^SET-EMD: Set-Theoretic Empirical Mode Decomposition

^o^HRV: Heart Rate Variability

^p^RR: Respiratory Rate

^q^SD: Standard Deviation

^r^STFT: Short-Time Fourier Transform

^s^MAE: Mean Absolute Error

^t^CI: Confidence Interval

## References

1. Wade EG, Fulton RM, Mackinnon J. The ballistocardiogram in the diagnosis of coronary arterial disease. Br Heart J. Jan 1956;18(1):65-77. [doi: 10.1136/hrt.18.1.65] [Medline: 13284187]

5. Smith DH. The ballistocardiogram as a predictor of myocardial infarction. Am J Cardiol. Feb 1959;3(2):247-249. [doi: 10.1016/0002-9149(59)90295-4] [Medline: 13626856]

9. Dinaburg AG, Zuckerman BR. Components of left ventricular dysfunction discriminated noninvasively beat by beat. Am J Cardiol. Nov 1, 1987;60(13):1123-1127. [doi: 10.1016/0002-9149(87)90365-1] [Medline: 3673911]

10. Dinaburg AG, Zuckerman BR. Left ventricular dysfunction discriminated noninvasively beat by beat. Am J Cardiol. Jan 1, 1984;53(1):238-242. [doi: 10.1016/0002-9149(84)90719-7] [Medline: 6691267]

11. Moser M, Pordy L, Chesky K, Taymor RC, Master AM. The ballistocardiogram in myocardial infarction: a study of one hundred cases. Circulation. Sep 1952;6(3):402-407. [doi: 10.1161/01.cir.6.3.402] [Medline: 14954536]

12. Kuo PT, Joyner CR Jr. Angina pectoris induced by fat ingestion in patients with coronary artery disease; ballistocardiographic and electrocardiographic findings. J Am Med Assoc. Jul 23, 1955;158(12):1008-1013. [doi: 10.1001/jama.1955.02960120008004] [Medline: 14392044]

13. Starr I, Pedersen E, Corbascio AN. The effect of nitroglycerine on the ballistocardiogram of persons with and without clinical evidence of coronary heart disease. Circulation. Oct 1955;12(4):588-603. [doi: 10.1161/01.cir.12.4.588] [Medline: 13261311]

14. Isaacs J, Wilburne M, Gunther L. Ultra-low frequency acceleration (force) ballistocardiogram in myocardial infarction. Am J Cardiol. Mar 1958;1(3):323-333. [doi: 10.1016/0002-9149(58)90297-2] [Medline: 13508554]

15. de Faire U, Theorell T. A simple ballistocardiographic measure (IJ/HI amplitude ratio) in relation to electrocardiographic evidence of ischaemic heart disease. Scand J Clin Lab Invest. Sep 1979;39(5):435-440. [doi: 10.3109/00365517909106128] [Medline: 574981]

16. Mandelbaum H, Mandelbaum RA. Studies utilizing the portable electromagnetic ballistocardiograph. IV. The clinical significance of serial ballistocardiograms following acute myocardial infarction. Circulation. Jun 1953;7(6):910-915. [doi: 10.1161/01.cir.7.6.910] [Medline: 13051833]

17. Smith JE. Comparison of the displacement, velocity, and acceleration ballistocardiography in coronary heart disease. Am Heart J. Nov 1953;46(5):692-704. [doi: 10.1016/0002-8703(53)90222-1] [Medline: 13104313]

18. Baker BM, Scarborough WR, Mason RE, Singewald ML. Coronary artery disease studied by ballistocardiography: a comparison of abnormal ballistocardiograms and electrocardiograms. Trans Am Clin Climatol Assoc. 1950;62:191-201. [Medline: 21407742]

19. Rivin AU. Clinical experience with the portable electromagnetic ballistocardiograph. Calif Med. Jan 1954;80(1):16-20. [Medline: 13116023]

20. Harrison WK, Talbot SA. Discrimination of the quantitative ultralow-frequency ballistocardiogram in coronary heart disease. Am Heart J. Jul 1967;74(1):80-87. [doi: 10.1016/0002-8703(67)90044-0] [Medline: 6027574]

21. Taymor RC, Pordy L, Chesky K, Moser M, Master AM. The ballistocardiogram in coronary artery disease. JAMA. Feb 9, 1952;148(6):419. [doi: 10.1001/jama.1952.02930060001001]

22. Scarborough WR, Mason RE, Davis FW Jr, Singewald ML, Baker BM Jr, Lore SA. A ballistocardiographic and electrocardiographic study of 328 patients with coronary artery disease; comparison with results from similar study of apparently normal persons. Am Heart J. Nov 1952;44(5):645-670. [doi: 10.1016/0002-8703(52)90095-1] [Medline:12985532]

23. Scarborough WR, Smith EW, Baker BM. Studies on subjects with and without coronary heart disease. Serum lipid, lipoprotein, and protein determinations and their relation to ballistocardiographic findings (a preliminary survey). Am Heart J. Jan 1960;59:19-35. [doi: 10.1016/0002-8703(60)90381-1] [Medline: 14442227]

24. Moss AJ. Ischemic heart disease and accelerated cardiovascular aging. A ballistocardiographic study. Circulation. Feb 1962;25:369-375. [doi: 10.1161/01.cir.25.2.369] [Medline: 14476526]

25. Mandelbaum H, Mandelbaum RA. Studies utilizing the portable electromagnetic ballistocardiograph. I. Abnormal HIJK patterns in hypertensive and coronary artery heart disease. Circulation. May 1951;3(5):663-673. [doi: 10.1161/01.cir.3.5.663] [Medline: 14831188]

26. Theorell T, Rahe CR. Life change events, ballistocardiography and coronary death. J Human Stress. Sep 1975;1(3):18-24. [doi: 10.1080/0097840X.1975.9939543] [Medline: 1235115]

27. Lynn TN, Wolf S. The prognostic significance of the ballistocardiogram in ischemic heart disease. Am Heart J. Sep 1974;88(3):277-280. [doi: 10.1016/0002-8703(74)90459-1] [Medline: 4855021]

28. Jones RJ. The Nickerson ballistocardiogram in arteriosclerotic heart disease with and without congestive failure. Circulation. Sep 1952;6(3):389-401. [doi: 10.1161/01.cir.6.3.389] [Medline: 14954535]

29. Ungerleider HE, Gubner R. Application of heart function tests in life insurance medicine. J Intern Med. Jan 12, 1953;145(s277):45-51. [doi: 10.1111/j.0954-6820.1953.tb04065.x]

30. Theorell T, Blunk D, Wolf S. Ballistocardiographic indicators of prognosis in ischemic heart disease. J Lab Clin Med. Jul 1975;86(1):46-56. [Medline: 1151142]

31. Bancroft WH, Swatzell RH, Baldone JC, Tucker MS, Eddleman EE. Computerized system for evaluation of coronary artery disease by noninvasive techniques. Presented at: ACMSE ’76: the 14th Annual ACM Southeast Regional Conference; Apr 22-24, 1976; Birmingham, Alabama. [doi: 10.1145/503636.503640]

32. Harrington N, Bui QM, Wei Z, et al. Passive longitudinal weight and cardiopulmonary monitoring in the home bed. Sci Rep. Dec 21, 2021;11(1):24376. [doi: 10.1038/s41598-021-03105-1] [Medline: 34934065]

33. Starr I, MacVaugh H III. Early and late effects of the coronary bypass operation on cardiac contractility and coordination. Am Heart J. Aug 1975;90(2):179-189. [doi: 10.1016/0002-8703(75)90118-0]

34. Cao X, Liu L, Cai D, Guo P, Tang J. Statistical analyses of ballistocardiogram features for cardiac disease diagnosis. J Tsinghua Univ. 2015;54:633-7.

35. Song Y, Ni H, Zhou X, Zhao W, Wang T. Extracting features for cardiovascular disease classification based on ballistocardiography. Presented at: 2015 IEEE 12th Intl Conf on Ubiquitous Intelligence and Computing, 2015 IEEE 12th Intl Conf on Autonomic and Trusted Computing and 2015 IEEE 15th Intl Conf on Scalable Computing and Communications and its Associated Workshops (UIC-ATC-ScalCom); Aug 10-14, 2015; Beijing. [doi: 10.1109/UICATC-ScalCom-CBDCom-IoP.2015.223]

36. Feng J, Huang W, Jiang J, et al. Non-invasive monitoring of cardiac function through ballistocardiogram: an algorithm integrating short-time Fourier transform and ensemble empirical mode decomposition. Front Physiol. 2023;14(1201722):37664434. [doi: 10.3389/fphys.2023.1201722]

37. RICHMAN SM, PRESCOTT R, LITTMANN D. Correlation of the ballistocardiogram with the coronary angiogram and electrocardiogram in ischemic heart disease. Am J Cardiol. Jan 1963;11(1):36-42. [doi: 10.1016/0002-9149(63)90028-6] [Medline: 13982157]

38. Scarborough WR, Penneys R, Thomas CB, Baker BM Jr, Mason RE. The cardiovascular effect of induced controlled anoxemia; a preliminary ballistocardiographic study of normal subjects and a few patients with suspected coronary artery disease. Circulation. Aug 1951;4(2):190-210. [doi: 10.1161/01.cir.4.2.190] [Medline: 14859396]

39. Henderson CB. Ballistocardiograms after cigarette smoking in health and in coronary heart disease. Br Heart J. Jul 1953;15(3):278-286. [doi: 10.1136/hrt.15.3.278] [Medline: 13059215]

40. Davis FW Jr, Scarborough WR, Mason RE, Singewald ML, Baker BM Jr. The effects of exercise and smoking on the electrocardiograms and ballistocardiograms of normal subjects and patients with coronary artery disease. Am Heart J. Oct 1953;46(4):529-542. [doi: 10.1016/0002-8703(53)90064-7] [Medline: 13092040]

41. Theorell T, Blunk D, Wolf S. Emotions and cardiac contractility as reflected in ballistocardiographic recordings. Pavlov J Biol Sci. 1974;9(2):65-75. [doi: 10.1007/BF03000526] [Medline: 4220366]

42. Mandelbaum H, Mandelbaum RA. Ballistocardiographic response to nitroglycerin in coronary artery disease. Am Heart J. Aug 1952;44(2):257-260. [doi: 10.1016/0002-8703(52)90150-6] [Medline: 14952446]

43. Jacobs HD. Ballistocardiographic observations. Br Heart J. Jan 1954;16(1):79-86. [doi: 10.1136/hrt.16.1.79] [Medline:13126361]

44. Trakhtenberg SB. The significance of some noninvasive methods in the objective evaluation of treatment of patients with coronary artery disease. Bibl Cardiol. 1976(35):197-201. [Medline: 825099]
