# Supplementary material for: Applications of Ballistocardiogram in the Diagnosis of Coronary Heart Disease: Systematic Review
Source: JMIR Cardio. 2025 Aug 8;9:e68197. doi: 10.2196/68197 (PMC12334112; doi:10.2196/68197)
Supplement: Checklist 1 [file cardio-v9-e68197-s003.docx]

| **Section and Topic** | **Item #** | **Checklist item** | **Location where item is reported** |
| --- | --- | --- | --- |
| **TITLE** | | |  |
| Title | 1 | Identify the report as a systematic review. | Page 1, Title |
| **ABSTRACT** | | |  |
| Abstract | 2 | See the PRISMA 2020 for Abstracts checklist. | Page 1–2, Structured Abstract (Background, Objectives, Methods, Results, Conclusions) |
| **INTRODUCTION** | | |  |
| Rationale | 3 | Describe the rationale for the review in the context of existing knowledge. | Page 3–4, Introduction |
| Objectives | 4 | Provide an explicit statement of the objective(s) or question(s) the review addresses. | Page 4, Introduction |
| **METHODS** | | |  |
| Eligibility criteria | 5 | Specify the inclusion and exclusion criteria for the review and how studies were grouped for the syntheses. | Page 5 “Selection Criteria”; Page 5–6 “Search Result” |
| Information sources | 6 | Specify all databases, registers, websites, organisations, reference lists and other sources searched or consulted to identify studies. Specify the date when each source was last searched or consulted. | Page 5 “Literature Search” |
| Search strategy | 7 | Present the full search strategies for all databases, registers and websites, including any filters and limits used. | Multimedia Appendix 2. Search Strategy. |
| Selection process | 8 | Specify the methods used to decide whether a study met the inclusion criteria of the review, including how many reviewers screened each record and each report retrieved, whether they worked independently, and if applicable, details of automation tools used in the process. | Page 5, Methods section – “Search Result”. |
| Data collection process | 9 | Specify the methods used to collect data from reports, including how many reviewers collected data from each report, whether they worked independently, any processes for obtaining or confirming data from study investigators, and if applicable, details of automation tools used in the process. | Page 6, Methods section – “Search Result”. |
| Data items | 10a | List and define all outcomes for which data were sought. Specify whether all results that were compatible with each outcome domain in each study were sought (e.g. for all measures, time points, analyses), and if not, the methods used to decide which results to collect. | Multimedia Appendix 1 |
|  | 10b | List and define all other variables for which data were sought (e.g. participant and intervention characteristics, funding sources). Describe any assumptions made about any missing or unclear information. | Multimedia Appendix 1; sample size marked as “-” if not reported in original article. |
| Study risk of bias assessment | 11 | Specify the methods used to assess risk of bias in the included studies, including details of the tool(s) used, how many reviewers assessed each study and whether they worked independently, and if applicable, details of automation tools used in the process. | Not reported in manuscript; no formal risk of bias assessment was performed. |
| Effect measures | 12 | Specify for each outcome the effect measure(s) (e.g. risk ratio, mean difference) used in the synthesis or presentation of results. | Multimedia Appendix 1; effect measures were heterogeneous and presented descriptively, including p-values (when available), percentages, and waveform characteristics.No standardized effect measures were used. |
| Synthesis methods | 13a | Describe the processes used to decide which studies were eligible for each synthesis (e.g. tabulating the study intervention characteristics and comparing against the planned groups for each synthesis (item #5)). | Methods section (Search Results, pages 5–6); Results section (narrative grouping, pages 7–15); Appendix 1 (chronological list). No formal synthesis groups. |
|  | 13b | Describe any methods required to prepare the data for presentation or synthesis, such as handling of missing summary statistics, or data conversions. | Not applicable; no data conversions or imputations were performed. All results were extracted and summarized as reported in the original studies. |
|  | 13c | Describe any methods used to tabulate or visually display results of individual studies and syntheses. | Multimedia Appendix 1 (tabulated summary of included studies, including author, year, country, content, sample, and results). |
|  | 13d | Describe any methods used to synthesize results and provide a rationale for the choice(s). If meta-analysis was performed, describe the model(s), method(s) to identify the presence and extent of statistical heterogeneity, and software package(s) used. | Methods section (pages 5–6): included studies grouped based on clinical context (ACS vs. non-ACS); results synthesized narratively; no meta-analysis was performed. |
|  | 13e | Describe any methods used to explore possible causes of heterogeneity among study results (e.g. subgroup analysis, meta-regression). | No methods to explore heterogeneity were performed. |
|  | 13f | Describe any sensitivity analyses conducted to assess robustness of the synthesized results. | Not reported; no sensitivity analysis was conducted. |
| Reporting bias assessment | 14 | Describe any methods used to assess risk of bias due to missing results in a synthesis (arising from reporting biases). | No methods for assessing reporting bias were applied. |
| Certainty assessment | 15 | Describe any methods used to assess certainty (or confidence) in the body of evidence for an outcome. | No certainty assessment methods were applied. |
| **RESULTS** | | |  |
| Study selection | 16a | Describe the results of the search and selection process, from the number of records identified in the search to the number of studies included in the review, ideally using a flow diagram. | Pages 5–7, Methods section; Figure 1 |
|  | 16b | Cite studies that might appear to meet the inclusion criteria, but which were excluded, and explain why they were excluded. | Page 7, Figure 1 |
| Study characteristics | 17 | Cite each included study and present its characteristics. | Pages 7–15, Results section; Multimedia Appendix 1 |
| Risk of bias in studies | 18 | Present assessments of risk of bias for each included study. | No formal risk of bias assessment was performed. |
| Results of individual studies | 19 | For all outcomes, present, for each study: (a) summary statistics for each group (where appropriate) and (b) an effect estimate and its precision (e.g. confidence/credible interval), ideally using structured tables or plots. | Pages 7–15, Results section; Multimedia Appendix 1(table of study characteristics and results) |
| Results of syntheses | 20a | For each synthesis, briefly summarise the characteristics and risk of bias among contributing studies. | No formal risk of bias assessment was performed. See Multimedia Appendix 1 for structured study characteristics. |
|  | 20b | Present results of all statistical syntheses conducted. If meta-analysis was done, present for each the summary estimate and its precision (e.g. confidence/credible interval) and measures of statistical heterogeneity. If comparing groups, describe the direction of the effect. | no meta-analysis or statistical synthesis was conducted. Results were narratively summarized in Results (pages 7–15) and Multimedia Appendix 1. |
|  | 20c | Present results of all investigations of possible causes of heterogeneity among study results. | No investigations of heterogeneity were conducted. |
|  | 20d | Present results of all sensitivity analyses conducted to assess the robustness of the synthesized results. | Not applicable; no sensitivity analyses were conducted. |
| Reporting biases | 21 | Present assessments of risk of bias due to missing results (arising from reporting biases) for each synthesis assessed. | Not applicable. |
| Certainty of evidence | 22 | Present assessments of certainty (or confidence) in the body of evidence for each outcome assessed. | Not reported in manuscript. No formal certainty assessment was conducted. |
| **DISCUSSION** | | |  |
| Discussion | 23a | Provide a general interpretation of the results in the context of other evidence. | Pages 16-18, Discussion |
|  | 23b | Discuss any limitations of the evidence included in the review. | Pages 17–18, in the Limitations section of the Discussion. |
|  | 23c | Discuss any limitations of the review processes used. | Not mentioned in the original manuscript, added in the revised version (page 17). |
|  | 23d | Discuss implications of the results for practice, policy, and future research. | Pages 16-18, Discussion. |
| **OTHER INFORMATION** | | |  |
| Registration and protocol | 24a | Provide registration information for the review, including register name and registration number, or state that the review was not registered. | No registration. |
|  | 24b | Indicate where the review protocol can be accessed, or state that a protocol was not prepared. | No protocol was prepared for this review. |
|  | 24c | Describe and explain any amendments to information provided at registration or in the protocol. | Not applicable – this review was not registered and no protocol was submitted. |
| Support | 25 | Describe sources of financial or non-financial support for the review, and the role of the funders or sponsors in the review. | Not applicable. |
| Competing interests | 26 | Declare any competing interests of review authors. | Conflicts of Interest section (to be added in the revised manuscript in page 19). |
| Availability of data, code and other materials | 27 | Report which of the following are publicly available and where they can be found: template data collection forms; data extracted from included studies; data used for all analyses; analytic code; any other materials used in the review. | Figure 1; Multimedia Appendix 1 |

*From:*  Page MJ, McKenzie JE, Bossuyt PM, Boutron I, Hoffmann TC, Mulrow CD, et al. The PRISMA 2020 statement: an updated guideline for reporting systematic reviews. BMJ 2021;372:n71. doi: 10.1136/bmj.n71. This work is licensed under CC BY 4.0. To view a copy of this license, visit <https://creativecommons.org/licenses/by/4.0/>
